# Lipoid Proteinosis in two Iranian Sisters: A Case Report and Review of Literature

**Published:** 2011-04-01

**Authors:** F Malekzad, H Rahimi, S Lotfi, M Qaisari

**Affiliations:** 1 Department of Dermatology, Skin Research Center, Shahid Beheshti University of Medical Sciences, Tehran, Iran

**Keywords:** Lipoid proteinosis, Urbach-Weithe disease, Hyalinosis cutis et mucosa

## Abstract

Lipoid proteinosis is a rare autosomal recessive disorder which may be seen within a family very occasionally. Herein, we report lipoid proteinosis in two sisters characterized by verrucous lesions and hoarseness of voice, dysphagia and multiple beaded papules along the margins of their eyelids, fissured lips and thick ferenulum.

## Introduction

Lipoid proteinosis is a rare autosomal recessive metabolic disturbance characterized by various cutaneous manifestations including hoarseness from early childhood and mucosal manifestations attributed to infiltrative deposits of oral cavity and uvula and some parts of upper respiratory tract. Extra-cutaneous features may include epilepsy, mental retardation and other neurologic and psychiatric manifestations.

Some cases have been reported from different sites of the world but lipoid proteinosis is very rare in siblings. Herein, we report two siblings with interesting verrucous lesions on limbs.

## Case Report

A 31 year-old Iranian woman presented with a long history of non-pruritic verrucous lesions involving the extensor of her limbs. She had a previous history of developing scars at sites of minor trauma and suffered from hoarseness. She had a history of several episodes of respiratory tract obstruction and dysphagia for two years and aphonia for four months. The patient had no history of epilepsy or photosensitivity.

Her older sister, aged 32, had similar but milder dermatological manifestations.

**Fig. 1:**
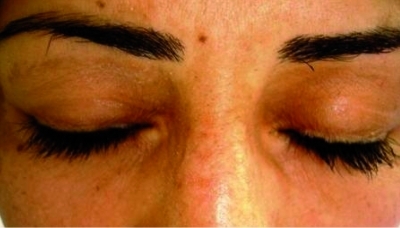
Flesh colored symmetric beaded papules on the eyelids

**Fig. 2:**
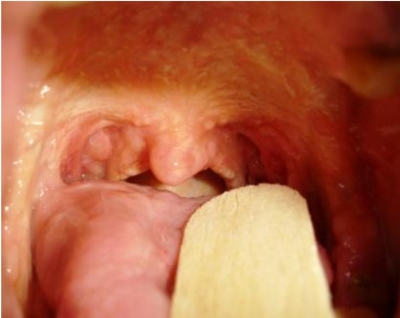
Yellow papules found on soft palate

On physical examination of these sisters, numerous flesh colored verrucous symmetric papules were observed on the elbows, knees, feet, toes and fingers. Yellow papules were found on soft palate (Figure 1) and multiple beaded papules along the margins of their eyelids (Figure 2). Multiple acneiform (pock like) scars were noticed over their forearms and legs. The younger sister had fissured lips and her frenulum was thickened and infiltrated and was unable to protrude her tongue out of the mouth. Other systemic examinations including central nervous system were normal. The routine hematological and biochemical investigations were within normal limits. Skull radiography was normal.

**Fig. 3:**
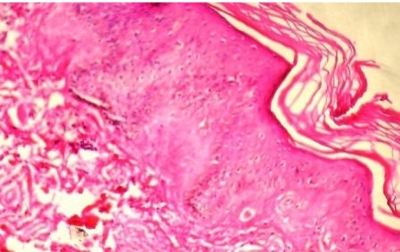
Deposition of a pink amorphous material in the papillary dermis and around blood vessels and appendages (H and E x10)

Histological examination of the skin lesions in the elbow showed deposition of a periodic acid-Schiffpositive (PAS +), pink amorphous material in the papillary dermis and around blood vessels and appendages (Figure 3). All these clinical and laboratory data were consistent with lipoid proteinosis.

## Discussion

Lipoid proteinosis was first described by Urbach and Weithe in 1929,[1] also called Urbach−Weithe disease or hyalinosis cutis et mucosa. It is an autosomal recessive disorder[2][3] characterized by persistent papules on the skin and mucous membranes.[4][5] This disease has a strong predilection for white races[3] with an increased incidence in Sweden and South Africa,[6] and has no sexual predilection.[3]

The first clinical sign is often hoarseness of voice, which presents at birth, or early childhood[2][4][5] and becomes prominent within the first few years of life and can progress to complete aphonia.[2] Mucosa of the lips, tongue and pharynx soon develop firm and yellow- white infiltrates. [2][4][5] The tongue is enlarged and becomes firm on palpation. [2][4][5] Skin changes become prominent in early life with the development of yellow-brown nodules on the face and lips.[2] Scattered lesions resembling atrophic, pitted, acne scars[2][4][5] may be seen on the face as well as on non-acne prone regions of the body.[4] Deposition of yellow materials induce a marked thickening of the facial skin with deep wrinkles, which may resemble solar elastosis.[4] Translucent keratotic papules are seen on the elbows and knees.[7] The skin shows increased susceptibility to injury from minor trauma and infection with recurrent attacks of impetigo, often bullous in nature.[8]

The eyelid lesions, which are pathognomnic for the disease, are described in 50% of cases.[3][8] These lesions appear as small, flesh colored papules seen along the margins of the upper and lower eyelid.[1][2] The appearance of these papules is variously described as 'string of beads' or 'eyelid beading'[9] and is also known as 'moniliform blepharosis'.[8] Characteristic bilateral calcifications or ossifications are found in temporal lobes[2] in 50-75% of cases.[8] Dental abnormalities are seen in 30% cases[4] and epilepsy may also be observed.[2]

The exact pathogenesis of this disease is not clear but has been postulated to be as a result of either a lysosomal storage disorder involving multiple enzyme defects or from a disturbance in collagen synthesis. [10][11][12] Recent studies have shown that it is the result of altered expression of exatracellular-matrixprotein-1 (ECM-1) gene.[10][13][14]

Histologically, lipoid proteinosis is characterized by deposition of a PAS-positive, diastase resistant material at the level of the basement membrane, papillary dermis, surrounding blood vessels, and around adnexal epithelia, especially sweat coils.[15][16] Ultrastructural examination reveals concentric rings of excess basement membrane surrounding blood vessels, and irregular reduplication of lamina densa at dermoepidermal junction resulting in an onion-skin appearance.[11]

Although more than 250 cases have been reported so far, occurrence of the disease in siblings is very rare. Interestingly, most familial cases have been reported from South Asia, including India,[17][18][19] Saudi Arabia,[20] Iran, [21] Kuwait[22] and Turkey,[23] where consanguineousmarriages are common.

Treatment of this condition is usually unsatisfactory. Reported approaches include oral steroids, dimethyl sulphoxide,[24] intralesional heparin, and etretinate.[10] CO2 laser surgery of vocal cords and beaded eyelid papules, and dermabrasion of skin result in cosmetic improvement. Except for the respiratory obstruction that occurs infrequently and rarely and requires tracheostomy, life expectancy is usually normal.[10]
